# Comparative Study by HPTLC of Selected *Capparis spinosa* Samples (Buds and Leaves) from the Cycladic Islands in Greece

**DOI:** 10.3390/foods14101827

**Published:** 2025-05-21

**Authors:** Evgenia Fotiadou, Antigoni Cheilari, Konstantia Graikou, Ioanna Chinou

**Affiliations:** Laboratory of Pharmacognosy and Chemistry of Natural Products, Department of Pharmacy, School of Health Sciences, National & Kapodistrian University of Athens, 15771 Athens, Greece; eugenia.fot@gmail.com (E.F.); cheilarianti@pharm.uoa.gr (A.C.); kgraikou@pharm.uoa.gr (K.G.)

**Keywords:** caper, Cyclades, rutin, quercetin, high performance thin-layer chromatography, total phenolic content, antioxidant activity, antimicrobial activity

## Abstract

*Capparis spinosa* is an edible plant with a long history in the Mediterranean region since antiquity. Its flower buds and leaves are mostly consumed salted or fermented (in vinegar) and are rarely eaten raw or dried. For the first time, caper samples subjected to different preservation processes (dried, salted, and desalted) were studied, foraged from the most producing Cycladic islands in Greece (Sifnos, Serifos, and Tinos). The quantitative determination of the flavonoids rutin and quercetin was carried out using high performance thin-layer chromatography (HPTLC), revealing the abundance of rutin in the buds and leaves (9.26–76.85 mg/g dry extract). Only one sample of desalted buds from Serifos showed a sufficient amount of quercetin (2.88 mg/g dry extract). The determination of total phenolic content (TPC) showed a decrease during brine (salted) preservation (11.7–37.7 mg GAE/g extract) compared to air-dried samples (50.9–62.4 mg GAE/g extract). The DPPH evaluation (8.0–35.2% inhibition at 200 μg/mL) was in agreement with the TPC results. All extracts showed stronger activity against Gram positive bacteria and the human pathogenic fungi *C. glabrata*. The samples from Sifnos exerted better bioactivities, with air-drying being the most effective preservation process in terms of antioxidant properties and phenolic content, although it resulted in a more bitter taste. Due to its high economic value, the caper holds great potential for further exploitation through better established and optimized processes in the food industry.

## 1. Introduction

*Capparis spinosa*, also known as the caper, is a spiny, prostrate, perennial shrub in the Capparaceae family. The genus *Capparis* includes about 150 species, many of which are endemic to tropical, subtropical, or temperate regions of the Mediterranean and Southeast Asia [1,2]. The plant is a xerophilous species demonstrating strong resistance to harsh environmental conditions and typically grows from April to May, ceasing its growth in September or October, depending on the climatic conditions of the region [3,4].

Capers have a long-standing and wide traditional use as a spice and food in Greece. Their flowers, buds (unripe flowers), leaves and, to a lesser extent, the fruits of wild plants are collected by the local population, particularly on the Cycladic islands. In some cases, the cultural significance of *Capparis* is further celebrated through local festivals, such as those held on Tinos Island, where capers are prominently featured in various culinary creations. Capers are preserved either by drying (in Sifnos), salting, or in vinegar brine (pickling) and serve as a key ingredient in various dishes, attributing a distinctive flavor that is slightly spicy and tangy. These products were traditionally traded through the cottage industry, and in recent years, they have been available on the market. Historically, *Capparis* has also been used for its medicinal properties, often prepared as herbal tea (roots and young shoots) against rheumatism and flatulence [5,6]. According to Dioscorides in *De Materia Medica*, the dry rind of the caper root was traditionally employed for its medicinal properties. It was believed to effectively cleanse persistent, hardened ulcers and was used in combination with vinegar to treat skin disorders such as leprosis. Additionally, the expressed juice of the plant was administered into the ears to eliminate parasitic worms [7]. There is limited literature on the Greek caper, with only two reports, one studying unripe flower buds in brine [8] and another by a Greek scientific group on leaves [9], without specifying the geographical area of collection.

According to the international literature, the species *C. spinosa* has been extensively studied and many of its metabolites have demonstrated biological activity, showing promising prospects for their use in the pharmaceutical and food supplement industries. In particular, *C. spinosa* is rich in phenolic compounds, flavonoids such as quercetin and kaempferol derivatives, alkaloids, fatty acids such as linoleic and oleic acid, sterols, and carotenoids [10]. Extracts of *C. spinosa* have been evaluated in vitro and in vivo, demonstrating strong antioxidant and anti-inflammatory activity, as well as analgesic and immunomodulatory properties [11]. In addition, the antimicrobial properties of *C. spinosa* have been assessed, with effects attributed to phenolic and flavonoid compounds. Moreover, the essential oil of *C. spinosa* has been characterized and exhibits various antimicrobial and insecticidal activities [12]. The traditional use of capers in treating diabetes has led to numerous in vitro and in vivo studies demonstrating its antidiabetic effects, as well as its nephroprotective and hepatoprotective properties [10,11,12]. The quantification of the major metabolites in *C. spinosa* has been achieved through various analytical techniques. High-performance liquid chromatography (HPLC), coupled with diode array detection (DAD) and mass spectrometry (MS), as well as quantitative Nuclear Magnetic Resonance (qNMR), have been employed to assess the concentration and profile of different compounds in both fresh and processed forms of capers [13,14,15]. HPTLC is an appealing alternative method for quantifying secondary metabolites in plant extracts due to its low cost, simplicity, accuracy, and robustness [16]. HPTLC has been utilized for the quantitative determination of quercetin and rutin in *Capparis zeylanica* Linn. [17] and *Capparis moonii* [18].

In the current study, samples of leaves and unripe flowers (dried, treated with brine (salted), and desalted) of the species *C. spinosa* subsp. *rupestris* var. *rupestris*—the identified caper species in the Aegean—were investigated. This subspecies is distinct from *C. spinosa* subsp. *spinosa* var. *aegyptia* (from the eastern Aegean islands), *C. spinosa* subsp. *spinosa* var. *canescens* (from Crete), and *C. spinosa* subsp. *rupestris* var. *rupestris* (from the Cyclades, Rhodes and Crete) [1]. The samples originated from three islands of the Cyclades (Sifnos, Serifos, and Tinos), where there is a large production and trade of capers, are studied for the first time. Initially, the chemical profile of all extracts was compared using HPTLC to evaluate the effect of brine processing on different dry parts. Additionally, the impact of desalination on the chemical composition of the samples was studied, as preservation in salt or vinegar is common in the food industry. Furthermore, rutin and its corresponding aglycon, quercetin, were quantified by means of HPTLC. Rutin and quercetin were selected for quantification based on the prevalence of rutin in *C. spinosa* as the most abundant flavonoid, especially in unripe flower buds and leaves. Quercetin, as the aglycone of rutin, is also of particular interest due to its potential release during salting via hydrolytic degradation of rutin glycosides. Moreover, both compounds are well-known for their strong antioxidant and antimicrobial activities, which align with the biological assays conducted in this study and hence, their presence serves as representative markers linking phytochemical content to biological activity. In addition, the total phenolic content (TPC), the free radical scavenging (DPPH), and the antimicrobial activity of the samples were evaluated. To the best of our knowledge, the chemical profiling of several caper samples from the Cyclades, the quantification of main flavonoids by HPTLC, and their biological evaluation is performed for the first time in the present investigation. This research will contribute to a better understanding of the chemical value and potential health benefits of capers and may encourage further investigations into this valuable Greek condiment that has been used for centuries by locals. Moreover, this study would also be beneficial as the recognition of capers from the Cycladic islands as of protected designation of origin (PDO) is ongoing from the Greek Ministry of Agriculture.

## 2. Materials and Methods

### 2.1. Plant Material and Extracts Preparation

Leaves and buds of *C. spinosa* subsp. *rupestris* var. *rupestris* were collected from three Cycladic islands (Sifnos, Serifos, and Tinos) during summer in 2021 and dried in the shade in a well-ventilated environment. The collection took place during the harvest period as indicated by the locals who collect capers traditionally on the islands during late spring-early summer when the buds are not blooming yet. Voucher specimens have been deposited at the Laboratory of Pharmacognosy and Natural Products Chemistry, National and Kapodistrian University of Athens. Moreover, buds and leaves preserved in brine (salt and water solution) were purchased from local farmers and half of the amount was left for desalination in water for 24 h, and all samples were dried in an oven at 40 °C for about 5 h and grinded by a laboratory mill (particle size ca. 1 mm). The dry samples were stored in darkness at room temperature and 5 g of each sample was extracted three times with methanol (250 mL) overnight at room temperature. The solvent was evaporated in vacuum to dryness on a rotary evaporator (Büchi Labortechnik AG, Flawil, Switzerland).

### 2.2. Solvents and Reagents

Analytical grade methanol (MeOH) for extraction, as well as acetonitrile (ACN), formic acid (FA), acetic acid (AA), sulfuric acid (H_2_SO_4_), vanillin, ethanol 96% (EtOH) for bioassays, and potassium hydroxide (KOH) were purchased from Merck (Merck, Darmstadt, Germany). Naturstoff reagent for HPTLC analysis was purchased from ThermoFisher (Thermo Fisher Scientific, Waltham, MA, USA). Distilled water was produced from LaboStar Pro TWF UV ultra-pure water system (Evoqua Water Technologies, Barsbuettel, Germany). For free radical scavenging and total phenolic content assays, Folin–Ciocalteu solution, dimethylsulfoxide (DMSO), sodium carbonate (Na_2_CO_3_), gallic acid, and 2,2-diphenyl-1-picrylhydrazyl (DPPH) were purchased from Sigma-Aldrich (Sigma-Aldrich, Steinheim, Germany). Rutin and quercetin were isolated as described by Ganos et al. [19] with sephadex LH-20 (25–100 μm, Pharmacia, Stockholm, Sweden) and for the quantification by HPTLC, rutin (Fluka™, Honeywell Specialty Chemicals, Seelze, Germany) and quercetin (Extrasynthese™, Genay, France) were purchased.

### 2.3. HPTLC Profiling and Quantification

For the analysis of samples and standards, 20 × 10 precoated silica gel 60 F_254_ and 60 RP-18 F_254_ plates were purchased from Merck (Germany). Automatic TLC Sampler-4 (CAMAG, Muttenz, Switzerland) was used to apply the samples and standards, band wise to the chromatographic plates, and development was accomplished in automatic development chamber (ADC2). The plates were sprayed with vanillin sulfuric acid reagent or Naturstoff reagent by CAMAG Derivatizer, scanned by TLC Visualizer 2 under white light, at 254 and 366 nm, and the chromatograms were processed by VisionCats 3.0 software.

Quantification analysis by HPTLC was carried out on normal (NP) and reverse phase (RP) plates (20 × 10 cm) where the band size of each track was 6 mm wide. The plates were developed in pre-saturated twin-trough glass chamber (20 × 10 cm) at room temperature using H_2_O/Acetonitrile/FA (65:35:1 *v*/*v*/*v*) for RP-HPTLC (quercetin quantification), and EtOAc/MeOH/H_2_O/FA (55:10:5:1 *v*/*v*/*v*) for NP-HPTLC analysis (rutin quantification). The developed and dried NP plates were quantitatively analyzed at 254 nm in absorbance mode, while the developed and dried RP plates were sprayed with Naturstoff reagent and analyzed at 366 nm.

### 2.4. Calibration and Validation

A concentration of 5 mg/mL in 90% MeOH was prepared for each sample and 20 μL were applied on the plates. Standard stocks of rutin and quercetin were prepared in 90% methanol, in concentrations 0.5 and 0.15 mg/mL, respectively. Different volumes of each stock solution were added to TLC plates, ranging from 0.6 to 20 μL and from 1 to 26 μL for rutin and quercetin, respectively. Thus, the range was 0.3–10 and 0.15–3.90 μg/band for rutin and quercetin, respectively. The method of validation was carried out as per the International Conference on Harmonization (ICH) guidelines for linearity range, accuracy, precision, repeatability, limit of detection (LOD), and limit of quantification (LOQ) [20].

The regression parameters, intercept, slope, and correlation coefficient (R^2^) were calculated by regression analysis, where y represents the peak area of the standard substance and x is the amount of this substance, as expressed by the peak area per spot (µg/spot). Calibration curves were obtained by plotting the peak areas of quercetin and rutin versus the concentrations of compounds HPTLC.

Accuracy was determined by recovery experiments spiking a sample with known amounts of rutin and quercetin. From analysis of the variability (triplicate analysis) of all recovery samples on 3 consecutive days, intra- and inter-day precision of the assay was assessed. The determination of LOD and LOQ for each analyte was calculated as LOD = 3.3 (SD/S) and LOQ = 10 (SD/S), respectively, where SD is the standard deviation of the y intercept and S the slope of the calibration curve.

### 2.5. Total Phenolic Content

The evaluation of the total phenolic content (TPC) of methanolic extracts was measured by the Folin-Ciocalteu reagent [21]. In a 96-well plate, 25 μL of each sample extract (4, 2, and 1 mg/mL) or a standard solution of gallic acid (2.5, 5, 10, 12.5, 20, 25, 40, 50, 80, and 100 g/mL) in dimethylsulfoxide (DMSO) was added to 125 μL of Folin-Ciocalteu solution (10%), followed by the addition of 100 μL of Na_2_CO_3_ solution. The plate was incubated for 30 min, in darkness, at room temperature. The absorbance at 765 nm was measured using a TECAN Infinite m200 PRO multimode reader (Tecan Group, Männedorf, Switzerland). All measurements were performed in triplicate, the mean values plotted on a gallic acid calibration curve, and the total phenolic acid content expressed as mg Equivalent to Gallic Acid (GAE) per gram of dry extract.

### 2.6. Antioxidant Activity

The free radical scavenging activity of the methanolic extracts was determined using the DPPH method [21] and tested at 3 different concentrations (200, 100, 50 μg/mL). Each extract (4, 2, and 1 mg/mL) was prepared using DMSO as a solvent. In a 96-well plate, 10 μL of each sample was mixed with 190 μL of DPPH solution (12.4 mg/100 mL in ethanol) and then incubated, at room temperature, for 30 min in dark room conditions. The absorbance was measured at 517 nm. All measurements were performed in triplicate and gallic acid was used as a positive control. The % inhibition of the DPPH radical for each dilution was calculated as: [(A − B) − (C − D)]/(A − B) × 100, where A: Control (w/o sample), B: Blank (w/o sample, w/o DPPH), C: sample, and D: Blank sample (w/o DPPH).

### 2.7. Antimicrobial Activity

All samples were investigated for their antimicrobial activity, against 8 strains of human pathogenic bacteria and 3 fungi by the disc dilution method on agar medium, evaluating the minimal inhibitory concentration (MIC). Specifically, they were tested against 4 Gram-positive bacteria: *Streptococcus mutans*, *Streptococcus viridans*, *Staphylococcus aureus* (ATCC 25923), and *Staphylococcus epidermidis* (ATCC 12228); 4 Gram-negative bacteria: *Escherichia coli* (ATCC 25922), *Enterobacter cloacae* (ATCC 13047), *Klebsiella pneumoniae* (ATCC 13883), and *Pseudomonas aeruginosa* (ATCC 227853); and 3 fungi: *Candida albicans* (ATCC 10231), *C. tropicalis* (ATCC 13801), and *C. glabrata* (ATCC 28838), of ATCC (American Type Culture Collection, Manassas, VA, USA).

The sterile 96-well polystyrene microtitrate plates were prepared by dispensing 100 μL of the appropriate dilution of the tested extracts in a broth medium, per well, in order to obtain the final concentrations of the tested extracts that ranged from 0.50 to 10 mg/mL. The inoculums that were prepared with fresh microbial cultures in sterile 0.85% NaCl, to match the turbidity of the 0.5 McFarland standard, were added to the wells to obtain a final density of 1.5 × 10^6^ CFU/mL for bacteria and 5 × 10^4^ CFU/mL for yeasts (CFU: colony forming units). After incubation (37 °C for 24 h), the MICs were assessed visually for the lowest concentration of the extracts, showing the complete growth inhibition of the reference microbial strains. An appropriate DMSO control (at a final concentration of 10%), a positive control (containing the inoculum without the tested samples), and the negative control (containing the tested derivatives without the inoculum) were included on each microplate. Standard antibiotic netilmicin (at concentrations of 4–88 μg/mL) was used to control the sensitivity of the tested bacteria and sanguinarine for oral bacteria, whilst 5-flucytocine (at concentrations of 0.5–25 μg/mL) and amphotericin B, were used as controls against the tested fungi (Sanofi, Diagnostics Pasteur at concentrations of 30, 15, and 10 μg/mL). For each experiment, any pure solvent used was also applied as a blind control. The experiments in all cases were repeated three times and the results were expressed as mean values.

### 2.8. Statistical Analysis

All experiments (TPC, TFC, DPPH radical scavenging, antimicrobial activity, and quantification) were performed in triplicate. Data were processed in EXEL, mean values and standard deviations were calculated with AVARAGE and STDEV functions. Relative standard deviations were calculated RSD: = (STDEV × 100)/AVERAGE value.

## 3. Results

### 3.1. Extraction

A total of 5 g of each *C. spinosa* sample was extracted with methanol and their yield (percentage %) was calculated. The dry weight of methanolic extracts and %yield is shown in Table 1.

### 3.2. HPTLC Analysis

**Figure 1:**
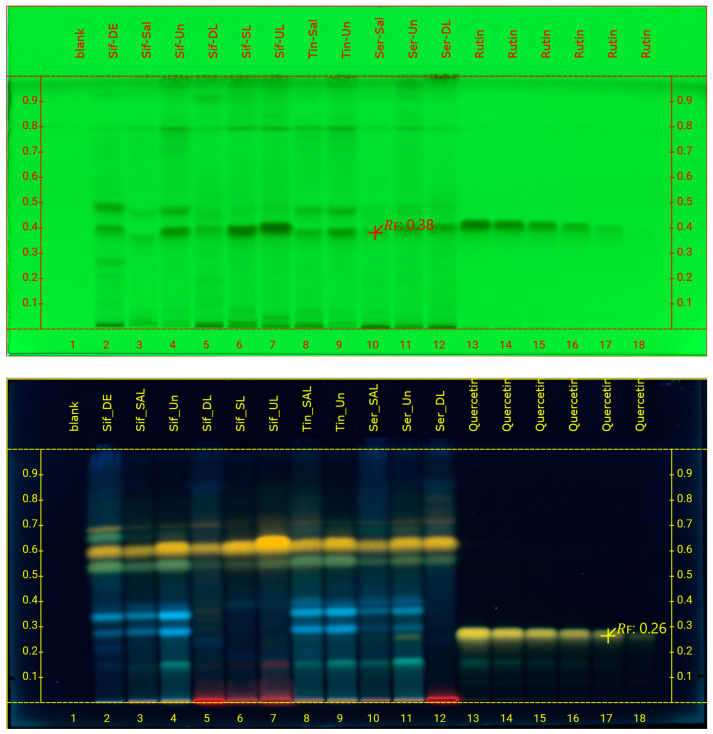
HPTLC chromatograms of the 11 caper samples and the 6 different amounts of the rutin standard solution after development of NP plates at 254 nm (**top**) and the quercetin standard solution after development of RP plates and derivatization with Naturstoff reagent, at 366 nm (**bottom**).

**Figure 2:**
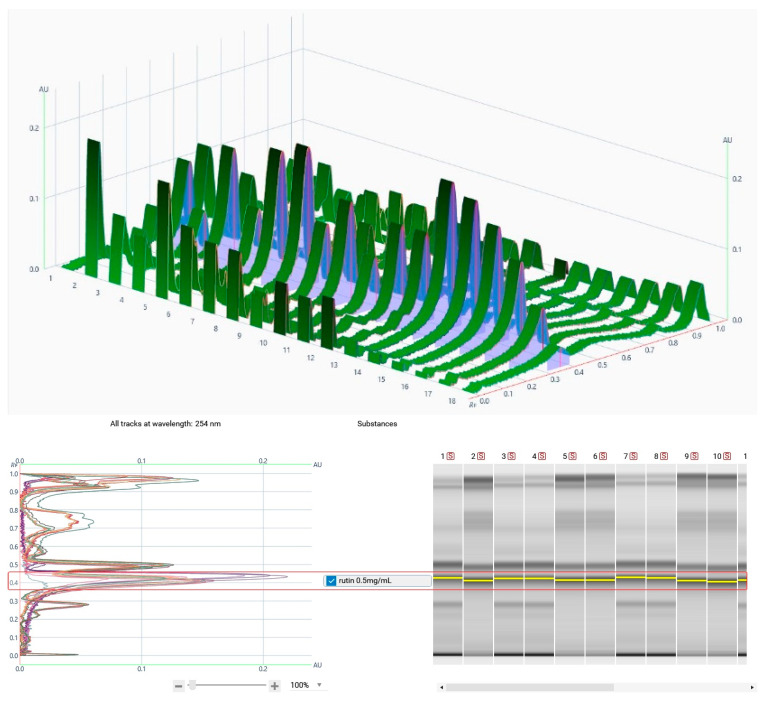
Example of HPTLC chromatograms generated from the plates by VisionCats software. (**Top**) Purple shading shows the Area Under the Curve (AUC) integrated for the calculation of the amount of targeted compounds (e.g., displayed for rutin, retention factor (Rf) 0.38) in the samples and standard solutions. (**Bottom**) Other view of HPTLC chromatogramms of all samples displayed in different colors and overlapped (**left**) and automatic peak detection (**right**).

For the quantification of rutin and quercetin, different TLC plates and developing solvent systems were tried. For quercetin, RP plates were developed with H_2_O/Acetonitrile/FA (65:35:1) solvent system, sprayed with Naturstoff reagent, and peak areas were analyzed at 366 nm. HPTLC chromatograms of the 11 caper samples and the 6 different amounts of the rutin and quercetin standard solutions are showed in Figure 1. For rutin, NP plates with EtOAc/MeOH/H_2_O/FA (55:10:5:1) as a mobile phase were selected for optimal separation, while the chromatograms for peak integration were observed at 254 nm (Figure 2).

The calibration curves were constructed from the known concentrations of the standards and the corresponding equations for rutin and quercetin were y = 0.0186x/(0.000003419 + x) + 0.0006548, R^2^ = 0.9999, and y = 0.06178*x/(0.000003443 + x) − 0.001701, R^2^ = 0.9996, respectively. The calibration curves were not forced to pass through the 0.0 point.

**Figure 3:**
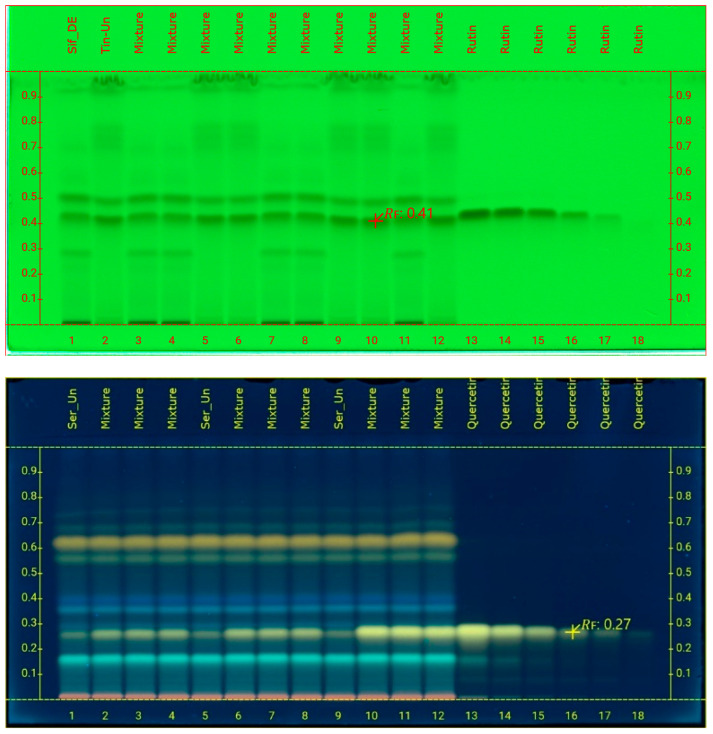
HPTLC chromatograms of the rutin spiking analysis at 254 nm, after development of NP plates (**top**) and quercetin spiking analysis, after development of RP plates and derivatization with Naturstoff reagent, at 366 nm (**bottom**). The amounts of rutin and quercetin added in the samples (low, medium, and high spiking) are shown in Table 3.

**Figure 4:**
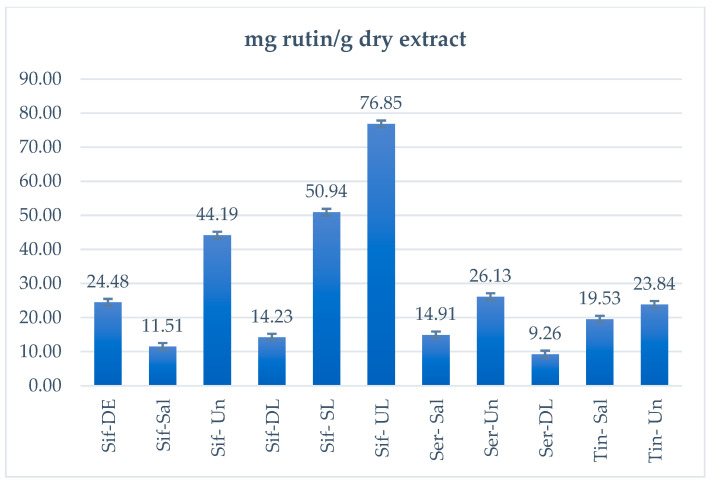
The amount of rutin per 1 g of dry extract for *Capparis spinosa* samples.

The LOQ and LOD for rutin were calculated 0.784 and 0.258 µg/band as well as 0.059 and 0.019 µg/band for rutin, respectively. The reference curves were checked for the accuracy of their own points to see if the model correctly predicted the theoretical concentrations. In particular, the maximum Er% was calculated as 4.04 and 5.67 for rutin and quercetin, respectively, and all other values were better. In addition, to calculate the %recovery of the method, new TLC plates were laid, where different amounts of standard rutin (0.25, 0.75, and 1.25 μg/band) were added to samples Sif-DE and Tin-Un, while amounts of quercetin (0.3, 0.45, and 1.95 μg/band) were added to Ser-Un (Figure 3). The %RSD of rutin was better than 2.8%, while it was better than 2.95% for quercetin. Finally, the amounts of rutin and quercetin in the samples are shown in Table 2 as well as in Figure 4 (rutin only). Quantification values were expressed as μg rutin/quercetin per band and mg rutin/quercetin per 1 g dry extract.

The % Recovery was greater than 82.16 ± 0.1 and 87.6 ± 1.18, for rutin and quercetin, respectively, with all values being within the acceptable limits, as shown in Table 3. It should be emphasized that for the samples preserved in brine, we do not have such a valid measurement, since the salted samples were simply drained and dried, resulting in the salt being fully counted in the weight of the dry extract, without the salt ratio being known.

### 3.3. Total Phenolic Content

The total phenolic content (TPC) of methanolic extracts was determined by the Folin-Ciocalteu method [21] and the results are shown in Table 4.

### 3.4. Antioxidant Activity

The methanolic extracts were tested for their antioxidant activity by their ability to inhibit the free radical DPPH in three different concentrations (200, 100, and 50 μg/mL) (Table 5).

### 3.5. Antimicrobial Activity

All the methanolic extracts were evaluated for their antimicrobial activity by dilution method against eight Gram-negative and -positive bacterial strains and three human-pathogenic fungi as shown in Table 6.

## 4. Discussion

Caper samples subjected to different preservation processes (dried, salted, and desalted) were studied, sourced from the most producing Cycladic islands in Greece (Sifnos, Serifos, and Tinos). Salted bud samples exhibited the highest extraction yield, ranging from 41.2 to 59.2%, affording almost twice the amount of extract of the corresponding desalted samples that ranged from 20.0 to 27.4%. Similarly, salted leaves afforded a 32.8% yield, while desalted samples yielded only 12.0%.

According to HPTLC results, the Ser-Un sample was the only one which contained a significant amount of quercetin (2.88 mg quercetin/g dry extract). All samples demonstrated a high rutin content, consistent with the existing literature supporting the abundance of rutin in various parts of *C. spinosa* [8,22,23,24,25]. Specifically, in untreated leaf samples from Iran, Mohebali et al. calculated 16.9 mg rutin/g dry weight [26], while another study from Iran found 0.61 mg rutin/g dry weight [27]. Proestos et al., in a leaf sample from Greece, measured rutin levels of 0.013mg/g dry weight [9]. Mollica et al. reported rutin levels of 19.3 mg/g dry extract in methanolic extracts of dry leaves from Italy [22]. Our results are in agreement with the literature [22,26], as in our dry leaf samples, rutin levels were calculated at 14.13 and 9.26 mg/g of dry extract from the islands of Sifnos and Serifos, respectively. However, our findings contradict the results by Proestos et al. [9], where they detected a hundred times less amount of rutin in samples of *C. spinosa* leaves (unspecified origin).

The experimental results for the sample of dry unripe flowers from Sifnos (Sif-DE) were consistent with the literature data and, in fact, this sample presented a greater amount of rutin (24.48 mg/g dry extract) than a sample from Italy, where rutin was calculated to be 18.9 mg/g dry extract [22]. Furthermore, it contained approximately four times the amount of rutin compared to a sample of dry unripe flowers from Turkey, which had 3.67 mg/g dry weight [28].

In the studied desalted samples of unripe flowers, rutin was calculated as 23.84, 26.13, and 44.19 mg/g dry extract for samples from Tinos, Serifos, and Sifnos, respectively. These results align with the existing literature, where rutin levels in desalted unripe flowers were reported at 26.2 mg/g dry extract [22].

In brine-treated unripe flower samples from Spain, Turkey, Morocco, Italy, and Greece, rutin concentrations have been recorded at approximately 1.1–3.1, 0.76–5.4, 0.8–10.47, 7.83, and 2.29 mg rutin/g extract, respectively [8]. In another study, on a sample preserved in brine from Turkey, rutin has been quantified at 3.16 mg/g dry extract [28]. In contrast, the brine-treated unripe flower samples analyzed in this study contained significantly higher rutin levels, specifically 11.51, 14.91, and 19.53 mg/g dry extract from Sifnos, Serifos, and Tinos, respectively. Notably, the sample of unripe flowers in brine from Tinos exhibited the highest amount of rutin, compared to those from Sifnos and Serifos.

In addition, Sifnos dry buds contained a higher rutin concentration (24.48 mg/g dry extract) compared to the dry leaf samples (14.23 mg/g extract). The samples in brine exhibited a lower concentration of rutin (11.51–19.51 mg/g extract) compared to the desalted ones (23.84–44.19 mg/g extract), as the presence of salt contributes to total sample weight. Additionally, according to the literature data, salt treatment can induce changes in chemical composition, such as the hydrolysis of flavonoid glycosides [25,28]. This may explain the decrease in rutin levels between the dry bud sample (Sif-DE, 24.48 mg/g dry extract) and the brine-preserved bud sample (Sif-Sal, 11.51 mg/g dry extract). However, this trend was not observed in the leaf samples (Sif-DL 14.23 mg/g dry extract and Sif-SL 50.94 mg/g dry extract). All samples of dry or processed unripe flowers and leaves, and especially the samples from Sifnos, contained significant amounts of rutin (~2448 and ~1423 mg rutin/100 g dry extract), confirming *C. spinosa* as a rich source of this bioactive compounds (onion: ~120 mg/100 g, thyme: ~2490 mg/100 g, and buckwheat: ~5350 mg/100 g) [8].

Moreover, the experimental results align with the findings of Inocencio et al. [8], who reported that the aglycon molecules of flavonoids, such as quercetin, are sensitive to oxidation and degrade over time, so they are usually only found in traces (0.03–1.5 mg/g in buds), with samples of buds in brine from Greece containing 0.54 mg/g quercetin [8]. Similar results were also shown by samples of dried and desalted buds from Italy, where no quercetin was detected in the 60% methanolic extracts [22]. Our findings on quercetin align with Inocencio et al., who report that under the influence of salt, hydrolysis of flavonoid glucosides leads to the formation of aglycone molecules, which, however, are sensitive to oxidation, resulting in their degradation and limited detection [8].

Overall, opinions differ and there is no clear consensus in the literature regarding the impact of salt preservation on chemical composition. Some researchers suggest that quercetin levels increase with preservation in brine [25,28], while Jiménez-López et al. argue that brining does not significantly affect phenolic compounds [29].

The total phenolic content (TPC) of the samples studied ranged from 11.7 to 62.4 mg GAE/g of dry extract, with the dry plant extracts exhibiting a higher phenolic content compared to the other preservation processes. In the samples of buds and leaves, a reduction of the TPC is observed when preserved in brine, compared to the dry samples. In addition, the desalted samples had a higher phenolic content than their corresponding brine-preserved samples. This discrepancy arises because the dry extract used for analysis contains salt, increasing the apparent weight while reducing the measured concentration of the active ingredients. The phenolic content was higher in the dry samples, with the samples from Sifnos showing the richest phenolic profile. In contrast, the unripe flower samples from Tinos exhibited the lowest TPC. Also, within the samples of Sifnos, it was observed that the dry leaves showed a higher phenolic content than the dry unripe flowers (buds), which agrees with the literature data for *C. spinosa* from other regions of the Mediterranean and Iran [26,30,31].

Preservation in brine has negative effects on the composition of the samples, leading to a reduction in TPC, as highlighted in several studies. Specifically, the fermentation process in brine causes hydrolysis of the flavonoid glycosides, as observed in the case of rutin, and oxidative degradation of the aglycon molecules [8]. This process also reduces the levels of glucosinolates and certain polyphenolic metabolites [32], such as epicatechin [25,28]. The literature reports indicate that the TPC of untreated unripe flowers from Turkey and Tunisia ranged from 17 to 38 mg GAE/g dry extract, whereas samples preserved in brine ranged from 6.5 to 11.0 mg GAE/g dry extract [28]. However, some researchers consider the difference between dry and processed fruit samples from Spain to be negligible [29].

Variations in the chemical composition of similarly processed samples can be attributed to differences in geographical origin, which influence environmental factors such as precipitation, temperature, altitude, geological conditions, and external variables. Therefore, these factors significantly affect the chemical profile of caper samples, as supported by the literature data [33].

A greater inhibitory activity against DPPH radical was observed in the dry leaves and unripe flowers from Sifnos, followed by the dry leaf extracts from Serifos. The weakest inhibition was recorded in the extract of unripe flowers preserved in brine from Tinos. In addition, the preservation of leaves and unripe flowers in brine appears to negatively impact on their antioxidant capacity, with a large difference in the inhibition rates compared to the dry samples. Specifically, the extracts of dry samples exhibited more than twice the DPPH inhibition compared to their brine-preserved counterparts. This finding aligns with a recent study from Turkey, which reported that preservation in brine reduces total phenolic content and consequently decreases antioxidant capacity by 30–52% [28]. However, some researchers argue that there is no significant difference in antioxidant activity between processed and untreated samples from Spain [29], while others suggest that fermentation can enhance antioxidant capacity due to the dissolution of cell walls and enzymatic activity [34]. This latter claim, however, is not supported by the findings of the present study. The abundant compound contributing to the antioxidant capacity of the samples appears to be rutin, which is the dominant metabolite in all *C. spinosa* samples. Several studies have highlighted its significant DPPH radical inhibitory activity [24,35,36,37].

Regarding antimicrobial activity, the samples exhibited stronger inhibition against Gram positive bacteria, including *S. aureus*, *S. epidermidis*, *S. mutans*, and *S. viridans*, while their effect on Gram negative ones (*P. aeruginosa*, *K. pneumoniae*, *E. cloacea*, and *E. coli*) was comparatively weaker. This difference is explained due to the structure of the cell wall where in Gram-positive bacteria, there is only a homogeneous thick peptidoglycan cell wall, while in Gram-negative bacteria there is a thinner layer of peptidoglycans—cell wall—but surrounded by a second outer membrane. Gram-positive bacteria lack this outer membrane, which is a natural barrier to potentially milder antimicrobials. In parallel, the samples exhibited strong antifungal activity against the human pathogenic fungus *C. glabrata*, while their effects on *C. albicans* and *C. tropicalis* were milder.

It is observed that the dry samples demonstrated better antimicrobial activity, with the dry leaf sample from Sifnos (Sif-DL) exhibiting the lowest MIC value, which agrees with the literature, highlighting the antimicrobial activity of dry leaves [38,39]. Conversely, the samples preserved in brine, especially the one from Tinos island, showed the weakest antimicrobial activity, likely due to the salt content in the sample weight, as previously discussed. In addition, the desalted samples showed stronger activity compared to the corresponding salted ones but remained less effective than the dry samples.

Notably, some samples with high TPC exhibited better antimicrobial activity. In particular, the dry samples, along with the desalted buds from Serifos (Ser-Un), which contained a rich phenolic profile, displayed the lowest MIC value. It is noteworthy that the desalted buds from Serifos (Ser-Un) were the only sample containing quercetin, which may have contributed to its potent antimicrobial activity. Furthermore, samples rich in rutin appeared to have a better antimicrobial profile and these findings align with the literature reports suggesting that antimicrobial activity is often attributed to phenolic compounds, particularly flavonoids [38,39].

## 5. Conclusions

The composition and biological activities of buds and leaves of *C. spinosa* subsp. *rupestris* var *rupestris* were studied for the first time in the present research, focusing on samples (dried, preserved in brine salted, and desalted) from the Cycladic islands of Sifnos, Serifos, and Tinos. These islands have the most extensive harvesting and culinary use of capers in Greece. In this study, two key flavonoids, rutin, and its aglycon molecule quercetin, were quantified using HPTLC. This method is widely recognized for establishing characteristic chemical fingerprints for natural products, its high accuracy (% recovery), sensitivity, and precision, with low LOD and LOQ for the quantification of both studied compounds. All samples of dry or processed unripe flowers and leaves, and especially the samples from Sifnos, contained significant amounts of rutin, confirming *C. spinosa* as a rich source of this bioactive compound. As for quercetin, while an increase in the amount was expected after the preservation in salt due to the hydrolysis of rutin, it was only detected and quantified in a single sample of buds from Serifos (2.88 mg quercetin/g dry extract). According to the literature, the hydrolysis of flavonoid glucosides leads to the formation of aglycone molecules during salting, which, however, are sensitive to oxidation, resulting in their degradation and limited detection. While this study did not include a targeted investigation on the degradation pathways of flavonoids under salt stress, the significantly lower levels of rutin and total phenolics in salted-preserved samples, compared to desalted and dried ones, strongly suggest that salt-related chemical transformations and analytical interference play a role in the observed differences. Future work could include controlled stability studies under different salting conditions, with and without antioxidant protection, to determine the precise effects of salting on flavonoid degradation kinetics. The quantification of rutin and quercetin by HPTLC allowed the comprehensive analysis of caper samples and has the potential to become a gold standard for the quantification of herbal drugs offering broader insights into the plant’s phytochemical composition. HPTLC proved to be cost-effective, required minimal solvent use and allowed simultaneous analysis of multiple samples from different producers of *C. spinosa* on the same plate. At this point, it is noteworthy to highlight the significant advantages of HPTLC in the quantification of flavonoids in plant extracts. HPTLC proved to be a reliable and efficient method for the separation and quantification of the targeted compounds, offering enhanced resolution, sensitivity, and reproducibility. In general, HPTLC is recognized in pharmacopeias for the quality control and standardization of herbal medicines and pharmaceutical products, making it suitable for large-scale screening and quality control [40,41]. These attributes made HPTLC a valuable tool for the accurate and precise quantification of flavonoids in caper as well as in other herbal products.

The antioxidant capacity of caper samples was in agreement with the total phenolic content, as the samples with the highest phenolic content exhibited higher inhibition of the DPPH radical and better antimicrobial activity. The samples studied demonstrated stronger antibacterial activity against Gram positive and mild activity against Gram negative strains. In parallel, the samples exhibited stronger antifungal activity against the human pathogenic fungus *C. glabrata*, and milder activity against *C. albicans* and *C. tropicalis*. The dry samples showed better antimicrobial activity, with the dry leaf sample from Sifnos showing the lowest MIC value.

Among all samples, the dried buds and leaves from Sifnos exhibited a higher total phenolic content, and interesting antioxidant and antimicrobial activity. In this respect, capers from Sifnos hold significant potential for further exploration. Regarding preservation methods, drying proved to be the most effective for retaining antioxidant compounds for edible use, despite the drawback of a slightly bitter taste. In conclusion, the caper, that has been utilized for centuries not only as a culinary ingredient but also for its medicinal properties, particularly in the Aegean islands where it thrives, represents a rich source of bioactive compounds with potent antioxidant and antimicrobial properties. Further investigations are needed to highlight their potential health benefits and applications in nutrition and medicine.

## Figures and Tables

**Table 1:** Dry weight of methanolic extracts and %yield of *Capparis spinosa* samples.

Sample	Code	Weight Of Methanolic Extracts (g)	%Yield
Sifnos dry buds	Sif-DE	2.69	53.8
Sifnos salted buds	Sif-Sal	2.06	41.2
Sifnos desalted buds	Sif-Un	1.00	20.0
Sifnos dry leaves	Sif-DL	0.90	18.0
Sifnos salted leaves	Sif-SL	1.64	32.8
Sifnos desalted leaves	Sif-UL	0.60	12.0
Serifos salted buds	Ser-Sal	2.69	53.8
Serifos desalted buds	Ser-Un	1.37	27.4
Serifos dry leaves	Ser-DL	0.96	19.2
Tinos salted buds	Tin-Sal	2.96	59.2
Tinos desalted buds	Tin-Un	1.33	26.6

**Table 2:** Quantification of rutin in the samples of *Capparis spinosa* measured as μg rutin/band ± standard deviation and expressed as mg rutin/g dry extracts.

Sample	μg Rutin/Band	SD	mg Rutin/g Dry Extract
Sif-DE	2.45	0.13	24.48
Sif-Sal	1.15	0.04	11.51
Sif-Un	4.42	0.23	44.19
Sif-DL	1.42	0.06	14.23
Sif-SL	5.09	0.26	50.94
Sif-UL	7.69	0.29	76.85
Ser-Sal	1.49	0.06	14.91
Ser-Un	2.61	0.14	26.13
Ser-DL	0.93	0.02	9.26
Tin-Sal	1.95	0.10	19.53
Tin-Un	2.38	0.13	23.84
**Sample**	**μg Quercetin/Band**	**SD**	**mg Quercetin/g Dry Extract**
Ser-Un	0.288	0.01	2.88

**Table 3:** % Recovery ± relative standard deviation of rutin and quercetin quantification in Capparis spinosa samples.

Chemical Compound	Sample	Added Amount per Band (μg)	HPTLC Recovery (% ± RSD)
rutin	Sif-DE	1.25	82.16 ± 0.10
		0.75	82.67 ± 0.30
		0.25	99.20 ± 0.48
	Tin-Un	1.25	90.24 ± 0.01
		0.75	92.60 ± 0.11
		0.25	97.60 ± 2.43
quercetin	Ser-Un	1.95	87.6 ± 1.18
		0.45	109.38 ± 0.52
		0.3	110.58 ± 2.27

**Table 4:** Total phenolic content ± standard deviation of Capparis spinosa samples expressed as mg GAE/g extract.

	Sample	TPC mg GAE/g Extract ± Standard Deviation
Buds	Sif-DE	58.3 ± 2.0
	Sif-Sal	18.6 ± 0.6
	Sif-Un	39.0 ± 2.8
	Ser-Sal	37.7 ± 2.1
	Ser-Un	48.8 ± 1.9
	Tin-Sal	11.7 ± 0.0
	Tin-Un	32.1 ± 0.9
Leaves	Sif-DL	62.4 ± 1.1
	Sif-SL	12.5 ± 2.3
	Sif-UL	27.4 ± 0.7
	Ser-DL	50.9 ± 1.4

**Table 5:** % Inhibition ± standard deviation of DPPH free radical of the *Capparis spinosa* samples in three concentrations.

	Sample	% Inhibition	SD	% Inhibition	SD	% Inhibition	SD
		200 μg/mL		100 μg/mL		50 μg/mL	
Buds	Sif-DE	31.4	1.1	18.5	0.2	10.2	0.9
	Sif-Sal	13.4	0.6	7.1	0.9	4.4	0.7
	Sif-Un	18.3	0.6	9.4	0.6	5.7	0.8
	Ser-Sal	16.7	0.6	10.4	0.6	5.7	0.5
	Ser-Un	26.0	0.5	15.7	2.5	8.5	0.6
	Tin-Sal	8.0	0.6	3.5	0.6	1.7	0.6
	Tin-Un	20.0	0.9	11.2	0.4	5.4	0.7
Leaves	Sif-DL	35.2	0.3	20.7	0.9	12.2	0.8
	Sif-SL	12.0	0.8	8.3	0.7	3.3	0.3
	Sif-UL	16.4	0.4	7.9	0.4	3.7	0.6
	Ser-DL	30.1	1.0	17.9	0.6	9.8	0.2

**Table 6:** Antimicrobial activity of Capparis spinosa samples, expressed as minimum inhibitory concentration MIC (mg/mL) ± standard deviation.

*Sample*	*S. aureus*	*S. epidermidis*	*P. aeruginosa*	*K. pneumoniae*	*E. cloacae*	*E. coli*	*S. mutans*	*S. viridans*	*C. albicans*	*C. tropicalis*	*C. glabrata*
*Sif-DE*	0.32 ± 0.04	0.27 ± 0.03	0.92 ± 0.07	0.98 ± 0.13	1.12 ± 0.01	0.92 ± 0.13	0.36 ± 0.02	0.30 ± 0.01	0.88 ± 0.06	0.86 ± 0.03	0.39 ± 0.02
*Sif-Sal*	0.67 ± 0.01	0.65 ± 0.07	1.32 ± 0.05	1.28 ± 0.12	1.44 ± 0.04	1.38 ± 0.09	0.68 ± 0.02	0.64 ± 0.02	1.00 ± 0.13	0.89 ± 0.09	0.71 ± 0.04
*Sif-Un*	0.49 ± 0.06	0.47 ± 0.07	1.00 ± 0.09	1.16 ± 0.09	1.32 ± 0.08	1.20 ± 0.07	0.52 ± 0.02	0.48 ± 0.02	0.99 ± 0.10	0.76 ± 0.04	0.56 ± 0.02
*Sif-DL*	0.28 ± 0.07	0.21 ± 0.03	0.88 ± 0.08	0.95 ± 0.1	1.00 ± 0.08	0.86 ± 0.10	0.32 ± 0.04	0.25 ± 0.01	0.88 ± 0,07	0.48 ± 0.01	0.38 ± 0.03
*Sif-SL*	0.88 ± 0.03	0.83 ± 0.05	1.45 ± 0.06	1.57 ± 0.09	1.52 ± 0.04	1.44 ± 0.10	0.89 ± 0.06	0.82 ± 0.02	1.37 ± 0.10	1.07 ± 0.08	0.91 ± 0.05
*Sif-UL*	0.59 ± 0.10	0.56 ± 0.06	1.14 ± 0.08	1.25 ± 0.08	1.39 ± 0.04	1.33 ± 0.11	0.64 ± 0.04	0.61 ± 0.02	0.96 ± 0.10	0.79 ± 0.05	0.65 ± 0.02
*Ser-Sal*	0.52 ± 0.09	0.50 ± 0.07	1.15 ± 0.07	1.18 ± 0.04	1.35 ± 0.04	1.26 ± 0.05	0.56 ± 0.03	0.51 ± 0.02	0.94 ± 0.09	0.84 ± 0.05	0.58 ± 0.01
*Ser-Un*	0.39 ± 0.03	0.37 ± 0.03	0.96 ± 0.13	1.12 ± 0.04	1.15 ± 0.08	0.99 ± 0.05	0.43 ± 0.03	0.39 ± 0.01	0.92 ± 0.04	0.69 ± 0.01	0.47 ± 0.01
*Ser-DL*	0.36 ± 0.04	0.38 ± 0.01	0.98 ± 0.09	1.07 ± 0.05	1.10 ± 0.08	0.94 ± 0.06	0.40 ± 0.01	0.34 ± 0.04	0.85 ± 0.06	0.57 ± 0.02	0.43 ± 0.02
*Tin-Sal*	0.97 ± 0.13	0.93 ± 0.12	1.67 ± 0.08	1.59 ± 0.07	1.58 ± 0.11	1.49 ± 0.04	0.96 ± 0.02	0.90 ± 0.09	1.25 ± 0.07	1.12 ± 0.05	0.98 ± 0.05
*Tin-Un*	0.51 ± 0.06	0.49 ± 0.06	1.12 ± 0.08	1.19 ± 0.03	1.37 ± 0.13	1.25 ± 0.09	0.54 ± 0.01	0.50 ± 0.04	0.89 ± 0.10	0.65 ± 0.01	0.38 ± 0.01
*netilmicin*	3.75 × 10^−3^ ± 0.02	3.90 × 10^−3^ ± 0.03	7.25 × 10^−3^ ± 0.01	7.98 × 10^−3^ ± 0.02	7 × 10^−3^ ± 0.01	345 × 10^−3^ ± 0.03	-	-	-	-	-
*sanguinarine*							0.016 ± 0.03	0.012 ± 0.01	-	-	-
*5-flucytosine*	-	-	-	-	-	-	-	-	0.15 × 10^−3^ ± 0.03	0.94 × 10^−3^ ± 0.01	9.82 × 10^−3^ ± 0.02
*amphotericin B*	-	-	-	-	-	-	-	-	1.23 × 10^−3^ ± 0.02	0.43 × 10^−3^ ± 0.01	0.46 × 10^−3^ ± 0.02

## Data Availability

The original contributions presented in this study are included in the article. Further inquiries can be directed to the corresponding author.
